# A Model for How Signal Duration Can Determine Distinct Outcomes of Gene Transcription Programs

**DOI:** 10.1371/journal.pone.0033018

**Published:** 2012-03-13

**Authors:** Kevin D. Fowler, Vijay K. Kuchroo, Arup K. Chakraborty

**Affiliations:** 1 Department of Chemical Engineering, Massachusetts Institute of Technology, Cambridge, Massachusetts, United States of America; 2 Department of Chemistry, Massachusetts Institute of Technology, Cambridge, Massachusetts, United States of America; 3 Department of Biological Engineering, Massachusetts Institute of Technology, Cambridge, Massachusetts, United States of America; 4 Ragon Institute of MGH, MIT, and Harvard, Boston, Massachusetts, United States of America; 5 Center for Neurologic Diseases, Harvard Medical School, Brigham and Women's Hospital, Boston, Massachusetts, United States of America; National Jewish Health and University of Colorado School of Medicine, United States of America

## Abstract

The reason why IL-6 induces a pro-inflammatory response, while IL-10 induces an anti-inflammatory response, despite both cytokines activating the same transcription factor, STAT3, is not well understood. It is known that IL-6 induces a transient STAT3 signal and that IL-10 induces a sustained STAT3 signal due to the STAT3-induced inhibitor SOCS3's ability to bind to the IL-6R and not the IL-10R. We sought to develop a general transcriptional network that is capable of translating sustained signals into one response, while translating transient signals into a second response. The general structure of such a network is that the transcription factor STAT3 can induce both an inflammatory response and an anti-inflammatory response by inducing two different genes. The anti-inflammatory gene can bind to and inhibit the inflammatory gene's production and the inflammatory gene can bind to its own promoter and induce its own transcription in the absence of the signal. One prediction that can be made from such a network is that in SOCS3−/− mice, where IL-6 induces a sustained STAT3 signal, that IL-6 would act as an anti-inflammatory cytokine, which has indeed been observed experimentally in the literature.

## Introduction

In many examples in biology, a single transcription factor is able to activate multiple genes [1]–[3]. Also, different receptors are able to activate the same transcription factor, but each leads to unique outcomes [4]–[6]. How signal specificity is attained with all of this redundancy has long interested researchers. Manipulation of signal duration through the use of feedback loops, phosphatases, and ubiquitin ligases is one mechanism by which cells attain signal specificity when multiple receptors utilize the same pathway. For instance, both nerve growth factor (NGF) and epidermal growth factor (EGF) signal through the MAPK pathway, but NGF is able to generate a sustained ERK signal, while EGF generates a transient signal; sustained and transient signals in this case result in two different outcomes through the same pathway [6], [7]. It was recently discovered that NGF signaling leads to a positive feedback loop in the MAPK pathway, while EGF leads to a negative feedback loop, and this disparity accounts for the differences in signal duration [8]. One mechanism cells can use to translate signal duration into different outcomes at the gene transcription level is through manipulation of immediate early gene (IEG) stability [9]. ERK induces transcription of the IEG products and then can bind to and phosphorylate them. The phosphorylated form of the IEG is less susceptible to degradation allowing it to further assist in the activation of downstream programs. Transient signals are unable to induce the protected form of the IEG products and the signal degrades. The net result is that the signal propagates through the genetic network for sustained signals or dies out for transient signals.

A simpler example of two receptors sharing the same signaling pathway, but leading to two different outcomes, is the IL-6 and IL-10 receptor. Both receptors activate signal transducer and activator of transcription 3 (STAT3), which forms dimers upon phosphorylation and enters the nucleus where it acts as a transcription factor. Despite the simplicity of the pathway, the two receptors induce different cellular behaviors: IL-6 elicits a pro-inflammatory response, while IL-10 signaling leads to an anti-inflammatory response [10]. The anti-inflammatory response by IL-10 acts on a number of cell types including antigen presenting cells (APCs) and is characterized by the induction of gene products by STAT3, which inhibit the transcription of inflammatory genes [11]. One key distinction between the two pathways is that the inhibitor, suppressor of cytokine signaling 3 (SOCS3) induced by STAT3, can bind to the IL-6 receptor, but cannot bind to the IL-10 receptor. This leads to IL-6 inducing a transient STAT3 signal, while IL-10 induces a sustained STAT3 signal [10]. Two independent experiments demonstrated that blocking SOCS3's ability to inhibit IL-6 signaling leads to an anti-inflammatory response indistinguishable from that induced by IL-10 in macrophages [12], [13].

Recent work by Behar et. al. is an example of how mathematical modeling can be utilized to propose various network architectures in which cells can make decisions by signaling through multiple receptors with shared components, while retaining signal specificity [14], [15]. These models focus on signal events upstream of transcription. We sought to identify a simple transcriptional model that would allow cells to make decisions based entirely on the duration of the input signal to the transcriptional network. Developed in the context of how signal duration manipulation by SOCS3 can lead to different outcomes from the IL-6 and IL-10 signaling pathways, our model focuses on the choice whether to produce a generic inflammatory gene product I or a generic anti-inflammatory gene product A when activated by a common transcription factor pSTAT3.

## Methods

### Transcriptional network model development

**Figure 1:**
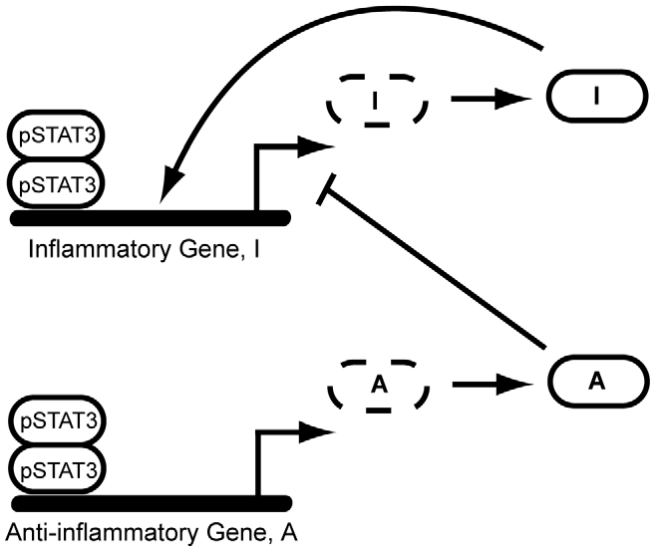
Transcriptional network that allows for cellular decisions based on signal duration. The “signal” in this mechanism is the ability of pSTAT3 to induce transcription of I or A. The cellular decision is based on the relative amounts of protein I or A produced. In this mechanism I can induce its own transcription in the absence of signal, pSTAT3. Protein A can bind to inflammatory gene I and inhibit its transcription.

A transcriptional network was formulated to allow the cellular decision of whether to be pro-inflammatory or anti-inflammatory to be made by changes in activated pSTAT3 signal duration. The network consists of two genes (I and A) that are activated by the same transcription factor (Figure 1). The essential feature of such a network must be that a transient pSTAT3 signal leads to dominant production of the inflammatory gene (I), while a sustained pSTAT3 signal leads to dominant production of the anti-inflammatory gene A. In reality, there are many inflammatory and anti-inflammatory genes, but we will adopt a coarse-grained view of the system where all of these genes will be characterized by generic representative genes I and A, respectively. In the model, which we shall refer to as Model 1, pSTAT3 dimers (pSTAT3) are allowed to bind to the promoter region of gene I (I_g_) and gene A (A_g_)
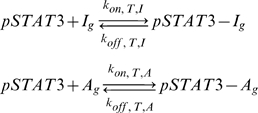
where the on and off rates can be different for the two genes. Different epigenetic modifications of the two genes could mean that one gene is more accessible than another, thus the possibility for different rates.

**Figure 2:**
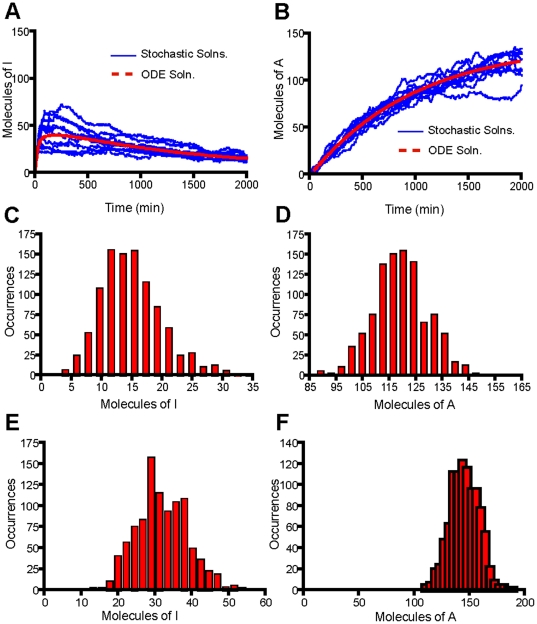
The transcription of A-molecules dominates for a sustained signal (K_deg,T_ = 0 min^−1^). (A) Time course trajectories for molecule I obtained by solution of the Model 1 equations using a mean field ODE solver (red curve) and ten times using the Gillespie Algorithm (10 blue curves). (B) Time course trajectories for molecule A obtained by solution of the Model 1 equations using a mean field ODE solver (red curve) and ten times using the Gillespie Algorithm (10 blue curves). (C) Histogram for molecule I obtained by solving the Model 1 equations using the Gillespie Algorithm 1000 times and recording the number of molecules at 2000 min. (D) Histogram for molecule A obtained by solving the Model 1 equations using the Gillespie Algorithm 1000 times and recording the number of molecules at 2000 min. (E) Histogram for molecule I obtained by solving the Model 2 equations using the Gillespie Algorithm 1000 times and recording the number of molecules at 2000 min. (F) Histogram for molecule A obtained by solving the Model 2 equations using the Gillespie Algorithm 1000 times and recording the number of molecules at 2000 min. Parameters for Model 1 were obtained from Table 1 and parameters for Model 2 were obtained from Table S2.

**Figure 3:**
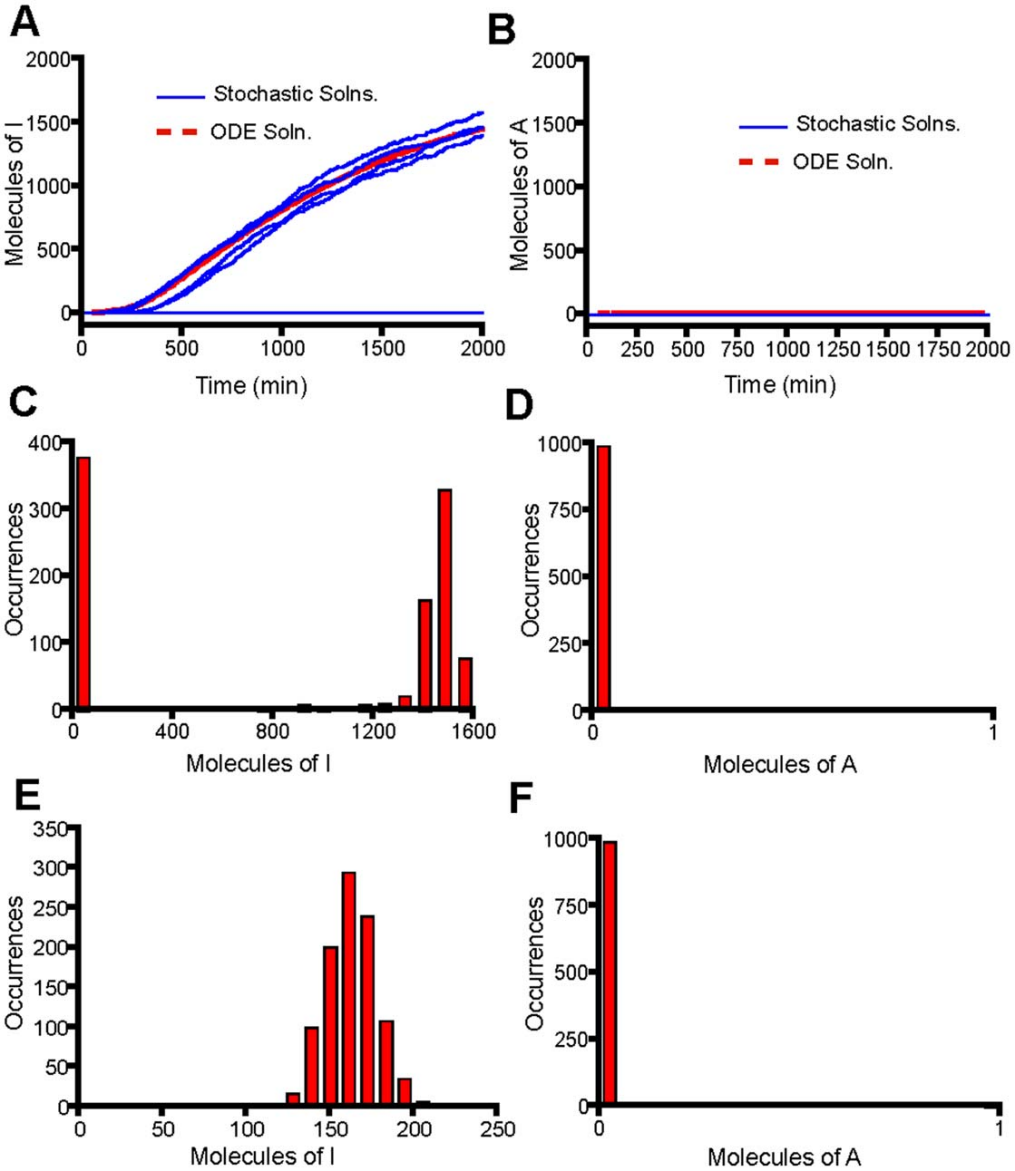
The transcription of I-molecules dominates for a transient signal (K_deg,T_ = 1.0 min^−1^ for Model 1 and K_deg,T_ = 0.1 min^−1^ for Model 2). (A) Time course trajectories for molecule I obtained by solution of the Model 1 equations using a mean field ODE solver (red curve) and ten times using the Gillespie Algorithm (10 blue curves). (B) Time course trajectories for molecule A obtained by solution of the Model 1 equations using a mean field ODE solver (red curve) and ten times using the Gillespie Algorithm (10 blue curves). (C) Histogram for molecule I obtained by solving the Model 1 equations using the Gillespie Algorithm 1000 times and recording the number of molecules at 2000 min. (D) Histogram for molecule A obtained by solving the Model 1 equations using the Gillespie Algorithm 1000 times and recording the number of molecules at 2000 min. (E) Histogram for molecule I obtained by solving the Model 2 equations using the Gillespie Algorithm 1000 times and recording the number of molecules at 2000 min. (F) Histogram for molecule A obtained by solving the Model 2 equations using the Gillespie Algorithm 1000 times and recording the number of molecules at 2000 min. Parameters for Model 1 were obtained from Table 1 and parameters for Model 2 were obtained from Table S2.

DNA-bound pSTAT3 can induce transcription of I mRNA (I_m_) and A mRNA (A_m_), via reactions assumed to be assumed first order
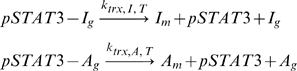
where the rate constants of the form k_trx,I,T_ should be read as the transcription of I in the presence of transcription factor, T (pSTAT3 dimer). It is assumed that the associated transcription factors dissociate each time a transcription reaction is carried out. Due to the simplicity of the network, it was easy to also construct a model where transcription factors do not dissociate each time mRNA is transcribed to verify that the qualitative behavior would not change, which we shall refer to as Model 2 (Web S1). We found multiple combinations of parameters that lead to the same qualitative behavior in both models (Figure 2, Figure 3, Web S1). For this reason, we shall mostly present results generated using Model 1 in the results section, unless otherwise noted. Any reference to “the model” is in reference to Model 1, where transcription factors dissociate after each transcriptional event.

**Table 1:** Table of parameters used in Model 1.

k_on,T,I_	0.015 molec^−1^ min^−1^	k_trx,I,I_	1.0 min^−1^
k_on,T,A_	0.015 molec^−1^ min^−1^	k_trx,I,A_	0 min^−1^
k_on,I,I_	0.015 molec^−1^ min^−1^	k_trl,I_	0.5 min^−1^
k_on,A,I_	0.015 molec^−1^ min^−1^	k_trl,A_	0.5 min^−1^
k_off,T,I_	0.5 min^−1^	k_deg,T_	0–10 min^−1^
k_off,T,A_	0.5 min^−1^	k_deg_	0.001 min^−1^
k_off,I,I_	0.5 min^−1^	k_deg,m_	0
k_off,A,I_	0.01 min^−1^	pSTAT3_o_	100 molec.
k_trx,I,T_	1.0 min^−1^	I_g_	2 molec.
k_trx,A,T_	0.1 min^−1^	A_g_	2 molec.

The parameters were selected in order to clearly demonstrate the phenomena of interest (i.e. I dominates transcription for transient signals while A dominates for sustained signals). The results presented were robust to a range of parameters as indicated by the sensitivity analysis (Web S1).

After transcription, the mRNA of I and A can be translated to fully functional proteins in assumed first order processes
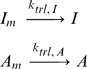
where species I and A represent the fully functional proteins.

The models also contain two types of feedback loops, which make the network sensitive to signal duration. Inflammatory protein I is allowed to bind to its own promoter and induce its own transcription in the presence or absence of pSTAT3 as given by
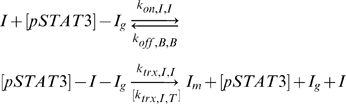
where [pSTAT3] indicates that pSTAT3 may or may not be present. If pSTAT3 is present, it is assumed that the rate of transcription is governed by whichever rate constant, k_trx,I,I_ or k_trx,I,T_, is greater. The ability of I to induce its own production in the absence of the signal through a positive feedback loop allows for the production of I even for a fast decaying signal as long as the decay rate of I is small enough to allow for the initiation of the positive feedback loop. Though we do not have direct experimental evidence for the existence of a positive feedback loop in this system, the addition of an autoregulatory positive feedback loop to our models is feasible since it has been identified as a general network motif [16], [17] and has been observed experimentally in other systems [18], [19]. The A gene has no positive feedback loops so it is entirely dependent on the signal and thus will not be produced in appreciable quantities by transient signals.

The network also contains a negative feedback loop where anti-inflammatory protein A is able to bind to the promoter of gene I and impede its transcription according to
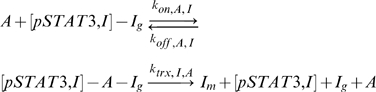
where [pSTAT3,I] indicates that either pSTAT3 or I is bound to gene I. The ability of A to negatively regulate I is important in creating a regime where A can win out. If the inhibition is strong enough (k_trx,I,A_<<0), A can shut down the production of I if A is present in great enough quantities. Sustained signaling may permit this scenario.

Another important aspect of the network is that all species, except genes, can decay at assumed first order rates
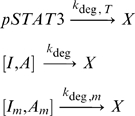
where [] represents either I or A. Biologically, the deactivation of I or A would be caused by ubiquitin ligases or phosphatases in the nucleus, which are assumed to be present in large enough concentrations so that the reactions are first order. The parameter, k_deg,T_, is a measure of the duration of the signal. Large values of k_deg,T_ indicate a highly transient signal, whereas small values k_deg,T_ indicate a relatively sustained signal. In the context of IL-6 and IL-10 signaling, k_deg,T_ is modified by the ability or inability of SOCS3 to inhibit pSTAT3 signaling. We begin the simulation just after appreciable amounts of SOCS3 have been induced, which means that the signal has reached its maximum value and then begins to decay at time zero. For IL-6, this occurs at about 30 minutes after signaling has begun [13]. Deactivation/degradation of I and A is allowed to occur whether or not the proteins are bound to genes. We also considered the case where deactivation/degradation of pSTAT3, I, and A is not allowed to occur when the molecules are bound to genes and found by sensitivity analysis that this does not significantly affect the qualitative results of the models (Web S1).

## Results and Discussion

### Genes I and A exhibit different responses to changes in signal duration

Rate laws were written for each of the reactions in the model and the corresponding chemical master equations were solved using the Gillespie algorithm [20]. We explicitly considered stochastic fluctuations in the number of molecules in this system via the Master equations because genes are often present in small copy numbers. The solution of the mean field equations could lead to the identification of signaling thresholds that in reality cannot be met due to fluctuations. For instance, the mean field solution to the Model 1 equations obtained via MATLAB predicts that the number of I molecules never reaches 50 molecules at any point in time for the set of parameters identified in Table 1 using a signal degradation rate of 0 (Figure 2A). However, the Gillespie algorithm shows that half of the trajectories of I cross 50; therefore it would be inaccurate to conclude that I never reaches the signaling threshold of 50 molecules (Figure 2A).

We are more interested in the general qualitative behavior of the genetic network as opposed to fitting the results to actual data, so no attempt was made to use experimentally derived parameters relevant to a specific system. As a result, it is important to use sensitivity analysis to verify that the qualitative results of the model are robust over a range of parameters. Sensitivity analysis was performed according to the procedures outlined in Web S1. The basic procedure was to divide all the parameters into nine different classes and testing all combinations of low, medium, and high values for each parameter class (Table S1). We then determined if the response could be switched from pro-inflammatory response to an anti-inflammatory response by changing the signal degradation rate for a given parameter set. In an initial search, 1809 parameter sets were identified that led to the desired qualitative behavior (Web S1).

A snapshot of the qualitative behavior of the model can be obtained by considering two values of k_deg,T_ with all other parameters fixed at the values given in Table 1. A value of 1 for k_deg,T_ corresponds to a transient signal, whereas a value of 0 for k_deg,T_ corresponds to a sustained signal. Sensitivity analysis revealed that the mRNA degradation rate did not alter the results (Figure S1), so the rate of degradation for I and A mRNA was set to 0. We consider the case where the rate of transcription of I is the same whether its induced by pSTAT3 or by itself (i.e. k_trx,I,T_ = k_trx,I,I_) and where the transcription of A is slower than the transcription of I (i.e. k_trx,A,T_<k_trx,I,T_, k_trx,I,I_). The qualitative results are robust to a range of parameters including the case where the rate of transcription of I and A is the same and the case where the rate of transcription of A is greater than I as determined by parameter sensitivity analysis (Web S1).

For sustained signals, the number of I molecules peaks at early times and then degrades, while the number of A molecules increases steadily through time (Figure 2A and Figure 2B). There are no negative feedback loops on A, and so, for sustained signals, the increase in its molecular levels is only hindered by its degradation. This is the cause for its steady increase with time. Conversely, I is strongly affected by the negative feedback loop at later times. Protein A is in high concentration at later times and is statistically likely to be bound to both copies of the I genes. In the parameter range chosen, A is able to bind tightly to the I gene and shuts down its production at later times. Once I production is shut down, the level of I gradually decreases due to its degradation/deactivation. The combined effect of the negative feedback loop and the degradation/deactivation reaction causes the peak in the number of I molecules at early times (Figure 2A).

An alternative way of viewing the data is to run 1000 trajectories, determine the molecular levels of I and A at “long times” (time = 2000 min), and construct a histogram. For sustained signals, the distributions appear similar in shape; however, I is at much lower levels than A (Figure 2C and Figure 2D). Therefore, these cells would appear to be producing mostly protein A if assayed at long times. This implies that the cells have made the decision to activate the anti-inflammatory gene program for sustained signals, an idea which will be explored in more detail in the next section. The same qualitative results were obtained using Model 2 (Figure 2E and Figure 2F).

**Figure 4:**
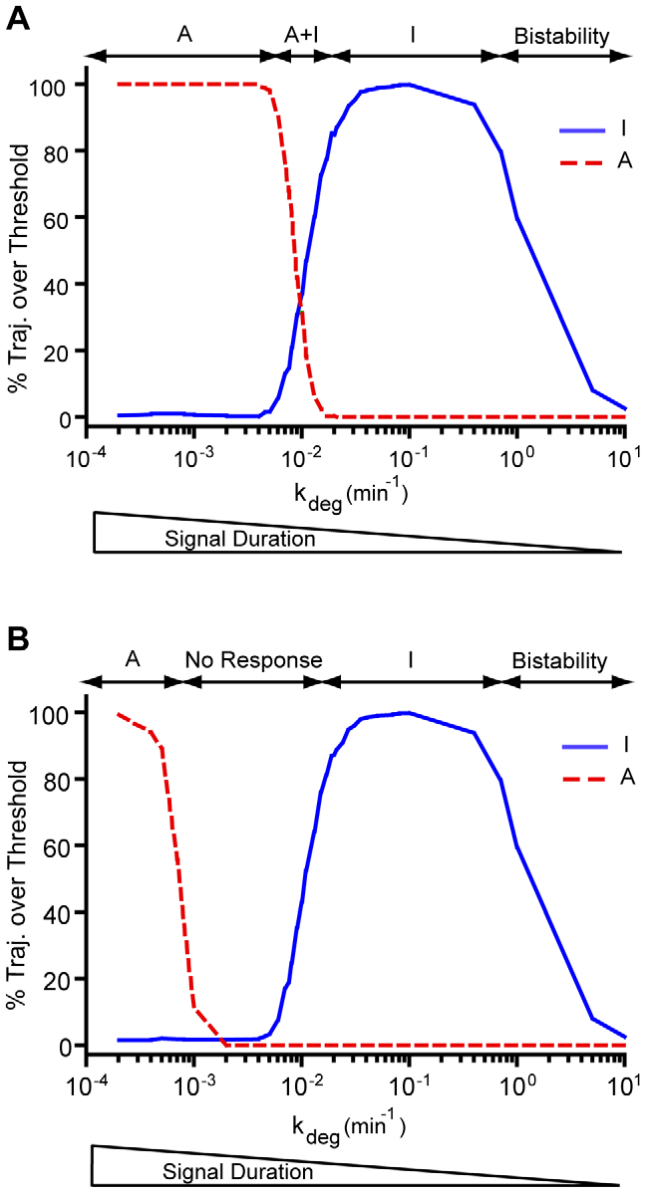
Cellular decisions can be made by manipulating signal duration. The maximum amount of I and A observed in 1000 time course trajectories measured out to 2000 min was compared to an arbitrary threshold and the percentage of trajectories crossing the threshold was computed for a wide range of signal degradation rates. (A) Different downstream thresholds were chosen for I and A such that both responses are observed in the intermediate crossover region. The I threshold was set to 100 molecules, while the A threshold was set to 20 molecules. (B) Using the same downstream threshold for both I and A leads to no response observed in the intermediate crossover region. The I and A thresholds were taken to be 90 molecules. The results were generated using Model 1 and the parameters were obtained from Table 1.

For transient signals, the molecular levels of I increase gradually with time in some of the trajectories, while A remains at or around zero in all of the trajectories (Figure 3A and Figure 3B). Protein A is entirely signal-dependent so it is not able to be produced in significant quantities when the signal degrades too quickly (Figure 3B and Figure 3D). As a result, the negative feedback loop is not effective for highly transient signals and the production of I is limited only by its ability to initiate the positive feedback loop. For this particular degradation rate, k_deg,T_ = 1, only some of the trajectories of I led to nonzero amounts, which is clearly observed in the long time histogram (Figure 3C). The bistability that results is entirely stochastic in nature, evident by the fact that the mean field time trajectory for I follows the average of the nonzero curves (Figure S2) [21]. Bistability results from the “roll of the dice” in determining whether or not the positive feedback loop is initiated before the signal and resulting I molecules degrade. It appears that bistability develops at approximately k_deg,T_ = 0.4 min^−1^ (Figure 4). As the signal becomes more transient in nature, an increasing number of I trajectories remain at I = 0. In many biological systems bistability serves as a tool in making cellular decisions, but in this system it is simply a consequence of the signal degrading too quickly and does not have direct relevance to the decision to be inflammatory versus anti-inflammatory [22]–[24]. Instead it determines what percentage of cells will be inflammatory or exhibit no response.

In silico knock-out experiments can be performed where either the positive or negative feedback loops are removed. When the positive feedback loop is removed, the results for sustained signals are virtually unchanged since the negative feedback loop dominates (Figure S3). For the transient case, both I and A would remain at approximately zero since both would be entirely signal dependent. The end result of blocking the positive feedback loop is that the cell would activate the anti-inflammatory gene program for sustained signals or neither program for transient signals. When the negative feedback loop is knocked out, the results for transient signals remain unchanged since the positive feedback loop dominates the behavior (Figure S4). For sustained signals, both I and A would be produced in significant quantities. There would no longer be a peak in I at short times, but a steady increase as a function of time. The end result of blocking the negative feedback loop is that the cell would choose the pro-inflammatory gene program for transient signals. The model predicts that both programs would be initiated for sustained signals, but this is likely not biologically relevant since the ability of anti-inflammatory genes to act “anti-inflammatory” has been removed by the deletion of the negative feedback loop. Therefore, sustained signals would likely lead to pro-inflammatory cells in this system. If both feedback loops were removed, both I and A would be entirely signal dependent and would exhibit similar qualitative behavior (Figure S5). The end result of blocking both the positive and negative feedback loops is that the cell would choose both I and A for sustained signals or neither for transient signals. Again, since the negative feedback has been deleted, it would likely be observed that the cells are pro-inflammatory for sustained signals or neither for transient signals.

The same qualitative results of A dominating transcription (Figure 2D and Figure 2F) over I (Figure 2C and Figure 2E) for sustained signals and I dominating transcription (Figure 3C and Figure 3E) over A (Figure 3D and Figure 3F) for transient signals is observed for both Model 1 and Model 2. Therefore, whether or not transcription factors dissociate after each transcriptional event is not important in this analysis. All the remaining results are based on the models allowing for either A or I to dominate transcription at different signal durations. We shall only consider results generated using Model 1, although the exact same conclusions would be reached using Model 2.

### Tuning signal duration as a tool for cellular decisions

From the two test values of k_deg,T_, it appears as if this mechanism could be a viable tool for making cellular decisions based entirely on signal duration modification. To further probe this idea, we constructed the following in silico experiment. Thousands of I and A trajectories similar to those obtained in the last section were generated for k_deg,T_ values ranging from 0 to 10 with all other parameters fixed at the values given in Table 1. It is assumed that the downstream gene programs (inflammatory or anti-inflammatory) activated by I or A are only initiated when the respective molecules cross a threshold value in molecular number at any point in the time trajectory. To measure this computationally, the maximum point in each trajectory was compared to a threshold value and the number of I and A trajectories crossing the threshold was recorded for each value of k_deg,T_. The percent of I or A trajectories crossing the threshold directly corresponds to the percent of cells that exhibit pro-inflammatory or anti-inflammatory responses in an equivalent biological experiment since each trajectory corresponds to 1 cell.

Cells with sustained signals (k_deg,T_<10^−3^) clearly exhibit an anti-inflammatory response while cells with transient signals (0.02<k_deg,T_<1) clearly exhibit an inflammatory response (Figure 4A and Figure 4B). There is a small crossover region for mildly transient signals (10^−3^<k_deg,T_<0.02), the behavior in which is determined by the choice in downstream threshold values for I and A. For the given set of parameters, it is clear that I has the capability of attaining a higher maximal value since its transcription rate is an order of magnitude higher than A and since I has a positive feedback loop (Figure 2B and Figure 3A). Therefore, one natural choice is to set the downstream threshold higher for I to account for this difference. With different I and A threshold values, the inflammatory and anti-inflammatory response curves overlap such that a mixture of cells exhibiting either response would be observed in the crossover region (Figure 4A). An alternative choice is to set the thresholds to be the same for both I and A, which leads to no response in the crossover region (Figure 4B). Setting a downstream threshold is an arbitrary process, which merely shifts the inflammatory and anti-inflammatory response curves, but does identify two distinct crossover region behaviors that may both have biological relevance.

One difficulty in performing this analysis is choosing where to set the long time cutoff for when an inflammatory or anti-inflammatory response can occur. For instance, it would not necessarily be biologically relevant if it takes several days of sustained signaling for A to cross its signaling threshold. For the purposes of this study, we arbitrarily chose this cutoff time to be 2000 minutes (∼1.5 days). Under most conditions, the decision is made much earlier than 2000 minutes, but we chose a longer time for inspection to ensure that we are identifying the decision for all k_deg,T_ values. As an example, consider Figure 3A and Figure 3B. For this k_deg,T_ value, the decision to be I and not A has occurred as early as 300 minutes. Given the nature of the curves, the same conclusion can be drawn by looking at 2000 minutes. Different parameter sets could give even earlier decision times or later decision times. Measuring at 2000 minutes allows us to reliably measure what decision has occurred, regardless of when it occurs. Immediate early gene transcription is on the order of minutes [25], while Th17 differentiation is on the order of 5 days [26]. Choosing 1.5 days as the cutoff seemed like a reasonable intermediate choice for considering biological relevance. An example of a cellular decision becoming evident after 1 day is in Th17 differentiation, where IL-17 mRNA starts to be transcribed in appreciable quantities at this time (unpublished observations).

Using 3.5 days as the cutoff time revealed an important limitation of our model. For any signal with a degradation rate greater than zero, I will eventually be produced in significant quantities if the simulation is allowed to run for long enough times (Figure S6A). The cause of this limitation is highlighted by the time course of I for a signal degradation rate of 0 (Figure 2). Even with a sustained signal, I does not go to zero since its potent positive feedback loop coupled with the sustained signal are still active in the moments when a given A molecule bound to the I gene dissociates or is deactivated, which can counteract the decrease in I due to its own degradation. For all cases where the signal degradation rate is not zero, A will eventually decay to zero since it is signal dependent. If the number of A molecules becomes low enough, it can no longer prevent the few remaining I molecules from initiating the positive feedback loop. Performing the threshold analysis at 3.5 days, I and A thresholds can still be chosen to allow for a cellular decision to be made based on signal duration changes, but the peak in I is broader and the crossover region occurs at smaller values of the signal degradation rate (Figure S6B). One way to deter this from occurring is to decrease the degradation rate of A, so that the negative feedback loop will remain active even at long times (Figure S6C). We do not believe that this limitation would be relevant under most biological situations since it would take longer than 3.5 days of steady signaling without any other changes within the cell for it to be an issue. Conceivably, once a pro-inflammatory or anti-inflammatory response was selected, the cell would have other means for shutting down the other set of genes. In this sense, the model is not entirely self-contained.

### Amplitude only significantly affects cellular decisions in the crossover region

Intracellular signals are generally described by two variables, signal amplitude and duration. Often in the literature, these two variables are combined into one quantity, the strength of signal. One interpretation of the strength of signal is the integrated signal, which is the area under the curve on a plot of signal strength vs. time. The gene transcriptional network depicted in Figure 1 was formulated to allow for disparate cellular decisions based strictly on differences in signal duration. To declare the cellular decisions made in the transcriptional model as strictly dependent on signal duration, it was also necessary to study the qualitative effect of modifying the signal amplitude.

**Figure 5:**
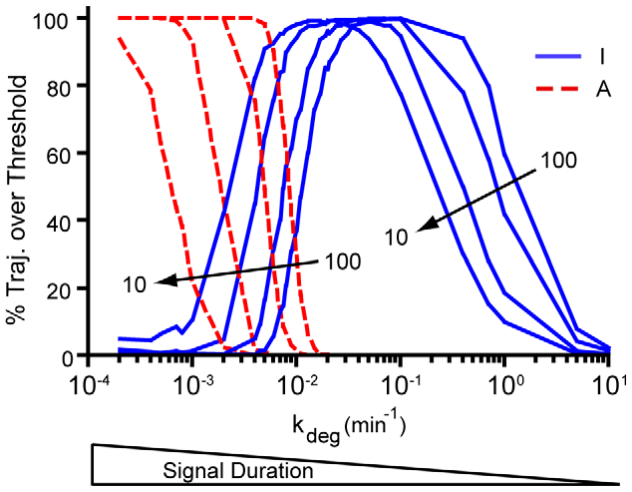
Altering the initial signal amplitude shifts the I and A curves, but does not affect their shape. The four sets of curves were generated using initial concentrations of 10, 20, 50, and 100 molecules of pSTAT3, respectively. For each initial concentration, the maximum amount of I and A observed in 1000 time course trajectories measured out to 2000 min was compared to an arbitrary threshold and the percentage of trajectories crossing the threshold was computed for a wide range of signal degradation rates. The I threshold was set to 100 molecules, while the A threshold was set to 20 molecules. The results were generated using Model 1 and the parameters were obtained from Table 1 unless otherwise noted.

The effects of amplitude modification were determined by setting the initial value of pSTAT3 molecules to different amounts and using the same procedure that was used to generate Figure 4. We observed that decreasing the signal amplitude from 100 molecules of pSTAT3 to 10 molecules shifted the I and A curves to the left without significantly altering their shape (Figure 5). The effects of amplitude modification can be observed within the crossover region from the inflammatory to anti-inflammatory response in that a 10-fold decrease in signal amplitude would shift the crossover from occurring at a degradation rate of 0.01 min^−1^ to 0.001 min^−1^. The production of A is entirely signal dependent, so significant decreases in the amount of pSTAT3 molecules translates into significant decreases in the amount of A molecules produced during the lifetime of the signal. As the number of A molecules is reduced, I becomes less regulated and will eventually win out. The effect of reducing pSTAT3 on the number of A molecules produced during the lifetime of the signal can partially be compensated for by increasing the lifetime of the signal, i.e. decreasing k_deg,T_, which accounts for the shift in the curves to the left. The crossover region is susceptible to these effects since it is at the edges of the inflammatory and anti-inflammatory response regimes.

Amplitude modification also affects significantly transient signals (k_deg,T_>0.1 min^−1^) by shifting the region of bistability to lower k_deg,T_ values as amplitude is decreased. As an example, consider a k_deg,T_ value of 0.2 min^−1^. For a signal amplitude of 100 pSTAT3 molecules, 100% of cells assayed would exhibit an inflammatory response. With a 10-fold decrease in amplitude, 50% of cells would exhibit an inflammatory response, while the other 50% of cells would exhibit no response. Therefore, amplitude only affects the number of cells exhibiting a given response for transient signals, but does not affect the type of response. The shift in the bistable region is also due to the fact that a decrease in signal amplitude can be partially compensated for by increasing the lifetime of the signal. Highly transient, low amplitude signals do not lead to any response since they do not have enough strength to kick off the positive feedback loop. Signals that are moderately sustained (k_deg,T_<0.001 min^−1^) or moderately transient (0.1<k_deg,T_<0.01), are unaffected by these changes in amplitude.

Decreasing the signal amplitude to five or less leads to both inflammatory and anti-inflammatory responses for sustained signals in some of the trajectories, unless the threshold for an inflammatory response is increased from that used to generate Figure 5 (data not shown). Since the transcription rate of I is faster than A, I is able to initiate the positive feedback loop before appreciable amounts of A are produced in some of the trajectories. At later times when appreciable amounts of A are produced, the I signal shuts down but had already crossed the inflammatory response threshold leading to an observed inflammatory response starting at early times and also an anti-inflammatory response at later times.

### Model predicts that IL-6 will promote an anti-inflammatory response in SOCS−/− cells

Another key difference between the IL-10R and the IL-6R, besides their differing interactions with SOCS3, is that the IL-10R has two motifs for STAT3 activation while the gp130 subunit of the IL-6R has four, leading to the IL-6R inducing a higher amplitude STAT3 signal [13], [27], [28]. Given that IL-6 induces a higher amplitude signal than IL-10, one could conclude that strong signals induce an inflammatory response, while weak signals induce an anti-inflammatory response. In this definition of signal strength, amplitude is the defining feature. If an experiment were performed where SOCS3 was no longer able to inhibit IL-6 signaling, the high amplitude signal would be converted from a transient signal into a sustained signal, and thus would have an even higher signal strength than before. Thinking about this problem in terms of signal strength, one would predict that IL-6 would still induce an inflammatory response when SOCS3 inhibition has been removed.

A revealing experiment performed by Yasukawa et. al. in which they exposed SOCS3−/−macrophages to IL-6 and LPS and measured the concentration of two pro-inflammatory cytokines (TNF and IL-12(p40)) in the supernatant showed just the opposite [12]. LPS induces an inflammatory response in macrophages, so a decrease in inflammatory cytokine production would be considered anti-inflammatory. As the dosage of IL-6 was increased, the pro-inflammatory cytokine concentration dropped meaning that IL-6 was acting as an anti-inflammatory cytokine on the SOCS3−/− macrophages. This puzzling result cannot be explained by a signal strength model where signal strength is essentially just a measure of the amplitude of the signal.

Our model is capable of making a prediction for how IL-6 will act on SOCS3−/− macrophages by making very few assumptions. We assume that IL-6 induces a moderately transient pSTAT3 signal that can roughly be approximated by a k_deg,T_ value of 0.1 min^−1^ in WT macrophages. The second assumption is that IL-10 induces a slowly decaying pSTAT3 signal that can be approximated by a k_deg,T_ value of 0.003 min^−1^ in both SOCS3−/− and WT macrophages. IL-6 and IL-10 would conceivably induce similarly decaying pSTAT3 signals in SOC3−/− macrophages, so we can use the same k_deg,T_ value. In their experiment, Yasukawa et. al. measure concentrations of secreted inflammatory cytokines. In our model this translates to counting the percentage of trajectories (cells) that cross the imposed threshold value of I at any point for a set value of k_deg,T_, which is characteristic to the macrophage type and the type of cytokine added along with LPS. By carrying out this analysis for a variety of initial amplitudes, we can make a prediction about how the macrophages will respond to increasing dosages of either IL-6 or IL-10. Since LPS induces an inflammatory response on its own, it is assumed that 100% of the cells would be pro-inflammatory in the absence of IL-6 and IL-10. Therefore, until a large enough signal amplitude is reached where gene A can cause a drop in the number of trajectories which cross the threshold value of I due to the negative feedback loop, it is assumed that all the cells will remain pro-inflammatory.

**Figure 6:**
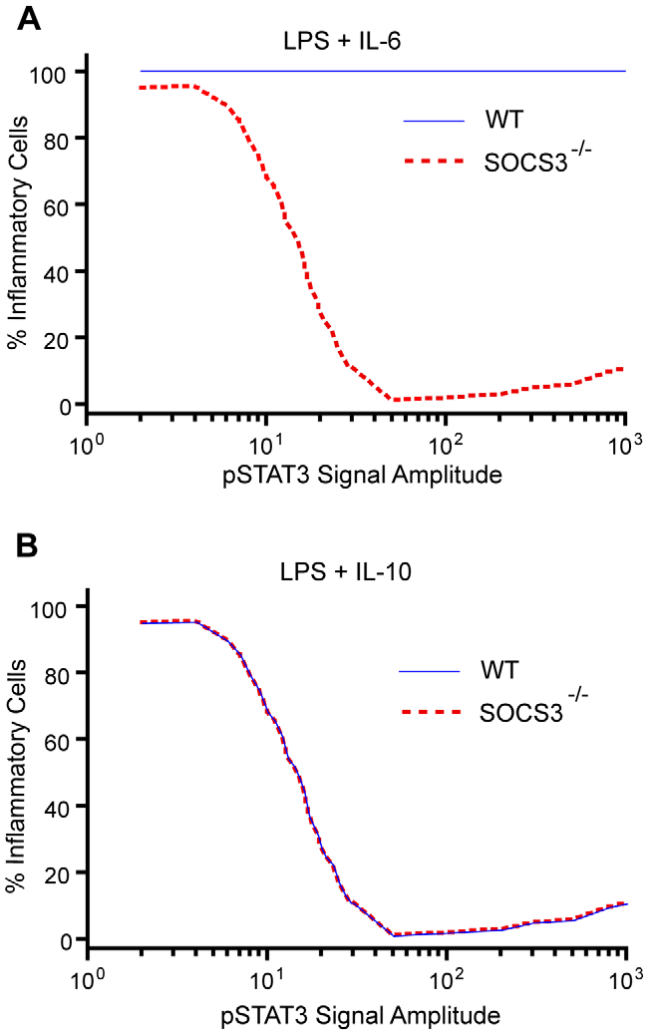
Model predicts that IL-6 will be anti-inflammatory in SOCS3−/− cells when given some inflammatory stimulus such as LPS for most pSTAT3 signal amplitudes. (A) Percentage of inflammatory cells predicted for transient pSTAT3 signals (WT) and sustained signals (SOCS3−/−) when the cells are exposed to LPS and IL-6. The value of k_deg,T_ used to represent the WT condition was 0.1 min^−1^, while a k_deg,T_ value of 0.003 min^−1^ was used to represent the SOCS3−/− condition. (B) Percentage of inflammatory cells predicted for sustained pSTAT3 signals (WT and SOCS3−/−) when the cells are exposed to LPS and IL-10. The value of k_deg,T_ used to represent these conditions was 0.003 min^−1^. For each initial concentration of pSTAT3, the maximum amount of I observed in 1000 time course trajectories measured out to 2000 min was recorded and the percentage of trajectories crossing the inflammatory threshold (100 I molecules) was determined for a value of k_deg,T_ corresponding to the signal duration expected for each condition. The results were generated using Model 1 and the parameters were obtained from Table 1 unless otherwise noted.

Using these assumptions, it is clear that the model predicts IL-6 will be anti-inflammatory when acting on SOCS3−/− macrophages (Figure 6). The model also captures the observed phenomenon that as the pSTAT3 amplitude is increased (i.e. the IL-6 dose is increased), IL-6 has a stronger anti-inflammatory effect. Since there is no distinction between IL-10 acting on SOCS3−/− or WT macrophages, the model predicts that no distinction will be seen experimentally, as was observed [12]. For transient signals, the model predicts a pro-inflammatory response, which is why the percentage of inflammatory cells remains at 100% for all amplitudes when WT macrophages are exposed to IL-6. If it were IL-6 alone, there would be a ramping up of the percentage of inflammatory cells at low amplitudes since a basal level of signaling is required to initial the positive feedback loop. However, since LPS induces an inflammatory response on its own, the ramping up period is not observed in the figure. There is no amplitude range for transient signals where A is capable of decreasing the percentage of inflammatory cells.

Yoshimura [10] has proposed that sustained STAT3 signals are essential for anti-inflammatory responses while transient signals promote inflammation, which is consistent with our model. The model serves as a tool for visualizing how the duration of the signal can lead to two different responses from the same genetic network activated by the same transcription factor from two different pathways. As further evidence of signal duration being a defining feature in determining the inflammatory response, El Kasmi et. al. also developed a system for analyzing this problem [13]. They transfected macrophages with an altered form of the EPO receptor (EPOR), which was capable of inducing pSTAT3 and was not susceptible to SOCS3 inhibition. This altered form of the EPOR induced an anti-inflammatory response indistinguishable from IL-10, which also supports the hypothesis that signal duration is a determinant of whether or not a response is inflammatory or anti-inflammatory [13].

While it is true that many different cytokines activate STAT3 that may or may not induce an inflammatory or anti-inflammatory response, often these cytokines activate multiple STAT molecules, such as STAT1 or STAT5, in addition to STAT3. It is beyond the scope of this analysis to determine how genes activated by other STATs affect the decision to be pro-inflammatory or anti-inflammatory. Since different STATs can be activated by the same receptor, heterodimer formation may be prevalent and it is difficult to predict how this may impact a network with this structure.

One could argue that the proposed transcriptional network model cannot easily account for common gene expression. The key wiring in our model that makes it sensitive to changes in signal duration is the positive and negative feedback loops. Common genes would not necessarily need to have the same wiring as the anti-inflammatory/inflammatory genes. As an example, consider the example of a common gene C that is also induced by pSTAT3. If this gene had a positive feedback loop, significant amounts of C would be produced from both transient and sustained signals. Even in the absence of a positive feedback loop, a transient signal still could produce significant amounts of C for high amplitude signals and low values of the C degradation/deactivation.

We have provided the structure of a gene transcriptional network which is sensitive to changes in signal duration. The essential feature of the model is that both genes of interest are activated by the same transcription factor and that one of the genes has a positive feedback loop to sustain itself in the absence of the main signal, but is also negatively regulated by the opposing gene. We believe that the model may be applicable to IL-6/IL-10 signaling since all essential steps of the model have been observed except for the positive feedback loop, namely that STAT3 induces both inflammatory and anti-inflammatory genes and that the anti-inflammatory genes inhibit the inflammatory genes. Experimentation would be necessary to identify a positive feedback loop in the inflammatory gene network in order for the model to be fully validated. Bioinformatic techniques could be used to determine whether inflammatory genes have the potential to bind to the promoter region of other inflammatory genes. Gene array analysis could then be used to verify the binding. Mutations to these promoter sites could then be used to knockout the positive feedback loop. Our model would then predict that IL-6 would not be able to induce an inflammatory response (Figure S3).

Even though the model was presented in the context of IL-6/IL-10 signaling, it may be applicable to a range of biological networks which are sensitive to signal duration. There has been extensive work in the field of systems biology to identify network motifs [16], [17]. Our network contains components of previously identified motifs such as an autoregulatory positive feedback loop [16]–[19] and a negative feedback loop. We are by no means the first to explore the effects of regulatory loops on cellular decision making. A number of studies have investigated the effects of positive feedback loops on decision making [24], [29]. Mangan and Alon described the characteristics and features of feed-forward loops [30]. Our focus in this work was not to propose a new signaling motif, but instead to show that a very simple genetic network could be used to explain the differences between IL-6 and IL-10 signaling without having to resort to using other more complicated arguments such as receptor-specific STAT3 conformational changes or effects due to unknown species. Further investigation is necessary to see how our model compares to existing models in the literature and to look for other examples in biology where our model may be applicable.

## Supporting Information

Web S1 **Discussion of sensitivity analysis.**
(DOC)

Figure S1 **Depiction of sensitivity analysis results.** Simulations were performed with all combinations of values (high, medium, low) for the nine different classes of parameters where the description of the classes and their respective high, medium, and low values are defined by Table S1. Each parameter set that led to a positive result (i.e. signal duration allows for a decision between I and A) was recorded. We computed for each of the 9 parameter classes, what percentage of these parameter sets had high values, medium values, or low values for the given parameter class. As an example, consider k_trx,I_. Out of all the parameter sets that led to a positive result, 71% had the high value for k_trx,I_, 29% had the medium value, and zero had the low value.
(JPG)

Figure S2 **Only the nonzero stochastic solution trajectories follow the mean field solution to the model equations for molecule I at a signal degradation rate of 1.0 min^−1^.** Time course trajectories obtained by solution of the Model 1 equations using a mean field ODE solver (red curve), ten times using the Gillespie Algorithm (10 blue curves), average at each time point of the nonzero stochastic trajectories (green curve), and average at each time point of all the stochastic trajectories (cyan curve). The results were generated using Model 1 and the parameters were obtained from Table 1.
(JPG)

Figure S3 **Upon deletion of the positive feedback loop, A is dominates transcription for sustained signals, while neither is significantly transcribed for transient signals.** Histogram for (A) molecule I and (B) molecule A obtained by solving the Model 1 equations for a sustained signal of K_deg,T_ equal to 0.0 min^−1^ using the Gillespie Algorithm 1000 times and recording the number of molecules at 2000 min. Histogram for (C) molecule I and (D) molecule A obtained by solving the Model 1 equations for a transient signal of K_deg,T_ equal to 1.0 min^−1^ using the Gillespie Algorithm 1000 times and recording the number of molecules at 2000 min. Parameters for Model 1 were obtained from Table 1.
(JPG)

Figure S4 **Upon deletion of the negative feedback loop, both I and A are strongly transcribed for sustained signals, while only I is significantly transcribed for transient signals.** Histogram for (A) molecule I and (B) molecule A obtained by solving the model equations for a sustained signal of K_deg,T_ equal to 0.0 min^−1^ using the Gillespie Algorithm 1000 times and recording the number of molecules at 2000 min. Histogram for (C) molecule I and (D) molecule A obtained by solving the Model 1 equations for a transient signal of K_deg,T_ equal to 1.0 min^−1^ using the Gillespie Algorithm 1000 times and recording the number of molecules at 2000 min. Parameters for the model were obtained from Table 1.
(JPG)

Figure S5 **Upon deletion of the positive and negative feedback loops, both I and A are produced in significant quantities after 2000 min for sustained signals, while neither is produced in significant quantities for transient signals.** Histogram for (A) molecule I and (B) molecule A obtained by solving the Model 1 equations for a sustained signal of K_deg,T_ equal to 0.0 min^−1^ using the Gillespie Algorithm 1000 times and recording the number of molecules at 2000 min. Histogram for (C) molecule I and (D) molecule A obtained by solving the Model 1 equations for a transient signal of K_deg,T_ equal to 1.0 min^−1^ using the Gillespie Algorithm 1000 times and recording the number of molecules at 2000 min. Parameters for the model were obtained from Table 1.
(JPG)

Figure S6 **At very long times the model breaks down and I dominates transcription for all nonzero signals.** This is due to the fact that A is entirely signal dependent, so once the signal is gone it also decays to zero rendering it incapable of inhibiting the production of I. Since I has not decayed to zero, its positive feedback loop allows it to ramp up production when A is incapable of inhibition. (A) Mean field solution for molecule I at a signal degradation rate of 0.002 min^−1^ plotted out to 5000 min. (B) The maximum amount of I and A observed in 1000 time course trajectories measured out to 5000 min was compared to an arbitrary threshold and the percentage of trajectories crossing the threshold was computed for a wide range of signal degradation rates. The I and A thresholds were set to 100 molecules. The results were generated using Model 1 and the parameters were obtained from Table 1, thus K_deg,A_ is equal to 0.001 min^−1^. (C) Reducing the degradation rate of A (K_deg,A_) to a value of 0.0001 min^−1^ counteracts the effect of measuring out to 5000 minutes. The I threshold was set to 100 molecules, while the A threshold was set to 20 molecules. Sampled trajectories were 5000 minutes long.
(JPG)

Table S1 **Listing of the parameter classes and their corresponding low, mid, and high values used in the sensitivity analysis.** Low, mid, and high values for each parameter were selected arbitrarily to either test over a range of orders of magnitude or to test at values around those used to obtain all the results in the main text (Table 1 parameter values).
(DOC)

Table S2 **Table of parameters used in Model 2.** This is the model where transcription factors do not dissociate after a transcriptional event. The parameters were selected in order to clearly demonstrate the phenomena of interest (i.e. I dominates transcription for transient signals while A dominates for sustained signals).
(DOC)

## References

[1] Müller MR, Rao A (2010). NFAT, immunity and cancer: a transcription factor comes of age.. Nat Rev Immunol.

[2] Li Q, Verma IM (2002). NF-κB regulation in the immune system.. Nat Rev Immunol.

[3] Boros J, Donaldson IJ, O'Donnell A, Odrowaz ZA, Zeef L (2009). Elucidation of the ELK1 target gene network reveals a role in the coordinate regulation of core components of the gene regulatory machinery.. Genome Res.

[4] Murray PJ (2007). The JAK-STAT signaling pathway: input and output integration.. J Immunol.

[5] Okkenhaug K, Vanhaesebroeck B (2003). PI3K in lymphocyte development, differentiation, and activation.. Nat Rev Immunol.

[6] Marshall CJ (1995). Specificity of receptor tyrosine kinase signaling: transient versus sustained extracellular signal-regulated kinase activation.. Cell.

[7] Kholodenko BN (2007). Untangling the signaling wires.. Nat Cell Biol.

[8] Santos SDM, Verveer P, Bastiaens PIH (2007). Growth factor-induced MAPK network topology shapes Erk response determining PC-12 cell fate.. Nat Cell Biol.

[9] Murphy LO, MacKeigan JP, Blenis J (2004). Molecular interpretation of ERK signal duration by immediate early gene products.. Mol Cell Biol.

[10] Yoshimura A, Naka T, Kubo M (2007). SOCS proteins, cytokine signaling and immune regulation.. Nat Rev Immunol.

[11] Murray PJ (2005). The primary mechanism of the IL-10-regulated anti-inflammatory response is to selectively inhibit transcription.. Proc Natl Acad Sci U S A.

[12] Yasukawa H, Ohishi M, Mori H, Murakami M, Chinen T (2003). IL-6 induces an anti-inflammatory response in the absence of SOCS3 in macrophages.. Nat Immunol.

[13] El Kasmi KC, Holst J, Coffre M, Mielke L, de Pauw A (2006). General nature of the STAT3-activated anti-inflammatory response.. J Immunol.

[14] Behar M, Dohlman HG, Elston TC (2007). Kinetic insulation as an effective mechanism for achieving pathway specificity in intracellular signaling networks.. Proc Natl Acad Sci U S A.

[15] Behar M, Hao N, Dohlman HG, Elston TC (2007). Mathematical and computational analysis of adaptation via feedback inhibition in signal transduction pathways.. Biophys J.

[16] Alon U (2007). Network motifs: theory and experimental approaches.. Nat Rev Genet.

[17] Milo R, Shen-Orr S, Itzkovitz S, Kashtan N, Chklovskii D (2002). Network motifs: simple building blocks of complex networks.. Science.

[18] Bateman E (1998). Autoregulation of eukaryotic transcription factors.. Prog Nucleic Acid Res Mol Biol.

[19] Becskei A, Seraphin B, Serrano L (2001). Positive feedback in eukaryotic gene networks: cell differentiation by graded to binary response conversion.. EMBO J.

[20] Gillespie DT (1977). Exact stochastic simulation of coupled chemical reactions.. J Phys Chem.

[21] Artyomov MN, Das J, Kardar M, Chakraborty AK (2007). Purely stochastic binary decisions in cell signaling models without underlying deterministic bistabilities.. Proc Natl Acad Sci U S A.

[22] McClean MN, Mody A, Broach JR, Ramanathan S (2007). Cross-talk and decision making in MAP kinase pathways.. Nat Gen.

[23] Losick R, Desplan C (2008). Stochasticity and cell fate.. Science.

[24] Das J, Ho M, Zikherman J, Govern C, Yang M (2009). Digital signaling and hysteresis characterize Ras activation in lymphoid cells.. Cell.

[25] Sun H, Charles CH, Lau LF, Tonks NK (1993). MKP-1 (3CH134), an immediate early gene product, is a dual specificity phosphatase that dephosphorylates MAP kinase in vivo.. Cell.

[26] Bettelli E, Carrier Y, Gao W, Korn T, Strom T (2006). Reciprocal developmental pathways for the generation of pathogenic effector Th17 and regulatory T cells.. Nature.

[27] Moore KW, de Waal Malefyt R, Coffman RL, O'Garra A (2001). Interleukin-10 and the interleukin-10 receptor.. Annu Rev Immunol.

[28] Kamimura D, Ishihara K, Hirano T (2003). IL-6 signal transduction and its physiological roles: the signal orchestration model.. Rev Physiol Biochem Pharmacol.

[29] Xiong W, Ferrell JE (2003). A positive-feedback-based bistable ‘memory module’ that governs a cell fate decision.. Nature.

[30] Mangan S, Alon U (2003). Structure and function of the feed-forward loop network motif.. Proc Natl Acad Sci U S A.

