# Supplementary material for: A Model for How Signal Duration Can Determine Distinct Outcomes of Gene Transcription Programs
Source: PLoS One. 2012 Mar 13;7(3):e33018. doi: 10.1371/journal.pone.0033018 (PMC3302786; doi:10.1371/journal.pone.0033018)
Supplement: Web S1 — Discussion of sensitivity analysis. (DOC) [file pone.0033018.s001.doc]

## Web S1

***Discussion of Sensitivity Analysis***

Most of the results presented in this paper were generated using Model 1 and the parameters listed in Table 1. Since this was just one parameter set, it was important to verify that the qualitative results of the model were the same for a wide set of parameters. The model contains less than 100 reactions so computation times for the Gillespie simulations were quite short. This allowed us to run a great number of simulations over a wide range of parameter sets and actually visualize the effect of varying parameters, which is not feasible for larger networks. Despite there being 91 possible reactions in the network, only 17 of the rate constants were unique, as indicated in Table 1. We further reduced this down to nine to make it feasible to test all possible combinations of three different values for each of the nine classes of parameters. The nine parameter classes along with the low, mid, and high values used for the sensitivity analysis of the parameters is given in Table S1.

The sensitivity analysis was performed by first generating all 19,683 combinations of the three values for each of the nine parameter classes. Each of these combinations was given a label of the form, LMHLMHLMH, which means parameter 1 (k_on_) had its low value, parameter 2 (k_off T,I_)had its mid value, etc. Twenty trials were run for each parameter set for three different values of signal degradation (K_deg,T_ = 0.001 min^-1^, 0.01 min^-1^, 0.1 min^-1^). In each individual trial, the program records the maximum value of I and A obtained at any point in the 2000 minute trajectory. At the end of each trial, these maximum values are compared to the previous trials, such that at the end of all twenty trials, the program records the highest and lowest maximum value of I and A obtained in any trial. Therefore, the output for each parameter set is I_max_, I_min_, A_max_, and A_min_ obtained at three different values of signal degradation (K_deg,T_ = 0.001 min^-1^, 0.01 min^-1^, 0.1 min^-1^). Using these 12 values, we can determine whether or not a cellular decision has been made. If I_min_ is greater than A_max_ for one signal degradation rate, and A_min_ is greater than I_max_ at another signal degradation rate, then a signal duration dependent decision has been made. It may be easier to think about this as a comparison of two distributions, I and A. For the A distribution to be greater than I, its lowest tail must be greater than the upper tail for I and vice versa. If a decision has been made, the parameter set is assigned a plus sign, and if no decision has been made, it is assigned a minus sign.

It is also possible for I to be bistable, which would mean that I_min_ would be 0 and using the above procedure it would have been given a minus. However, it is probable that I_max_ would be much greater than A_min_ as observed in the main text. For this reason, we also recorded which parameter sets led to bistable results for the two transient signals (K_deg,T_ = 0.001 min^-1^, 0.01 min^-1^). Whenever the positive feedback loop was initiated, it usually resulted in I values much greater than 50, so the test for bistability was whether I_min_ was 0 and I_max_ was much greater than 50. Parameter sets marked bistable may lead to a cellular decision, but it is difficult to test since I_min_ is 0.

The sensitivity analysis revealed that there were 1809 parameter sets that led to a cellular decision, 15529 that did not, and 2345 that were bistable. Even though there were many parameter sets that did not lead to the same qualitative results, we consider the 1809 positive results to indicate that this is a fairly robust qualitative phenomenon for this model considering the range of parameters tested. Also, bistable results may still lead to a cellular decision at lower values of the signal degradation rate. There are various methods for analyzing sensitivity results. We chose to focus our efforts on analyzing the positive results. Within the subset of positive results, we calculated the percentage that had either a low, mid, or high value for each parameter class (Figure S1). The sensitivity of the results to a given parameter can be inferred by the size of the spread in the percentages for that given parameter class. If all of the positive results had an equal spread of low, mid, and high parameter values for a given parameter class, then the results were insensitive to changes in that parameter. The three least sensitive parameter classes were k_deg,m_, k_trl_, and k_trx I,A_. The low sensitivity to k_deg,m_ when one of the values tested was 0 justifies setting this value to 0 in the simulations given in the main text. Clearly the most sensitive parameters are the transcription rates, and the low value for k_on_ and the high value for k_deg I,A_ were too extreme.

Some additional information can be obtained by analysis of the sensitivity results. Of the 1809 positive results, 80 had A transcription rates higher than I. Therefore, it is not required for I to have a higher transcription rate than A for this model to be sensitive to signal duration. One assumption used to make Model 1 in the main text was that the transcription factors dissociate from the gene after each transcription event. It also may be possible that the transcription factors remain bound and transcription can occur until they dissociate according to their respective k_off_ value. We created Model 2 to test whether or not this type of model would lead to the same qualitative results. The only equations from the model development section in the main text that are changed in Model 2 are the following which involve transcription:

All other equations in Model 2 are the same as Model 1. As a quick test, we ran the sensitivity analysis program with the parameter set in Table S1 and found that 1900 parameter sets led to positive results. This is actually greater than that observed from Model 1 so the uncertainty about whether transcription factors dissociate after each transcription event does not affect the qualitative results of the model. Another assumption made in the models presented in the main text was that molecules pSTAT3, I, and A were able to be deactivated while bound to genes I and A. We tested the effect of this assumption by creating a model similar to Model 1, only where the molecules were not allowed to be deactivated while bound to genes. Using this new model, we ran the sensitivity analysis program with the parameter set in Table S1 and found that 1939 parameter sets led to positive results, indicating that the qualitative results are not significantly affected by the nature of this assumption.
