# Supplementary figures and images for: A Model for How Signal Duration Can Determine Distinct Outcomes of Gene Transcription Programs
Source: PLoS One. 2012 Mar 13;7(3):e33018. doi: 10.1371/journal.pone.0033018 (PMC3302786; doi:10.1371/journal.pone.0033018)

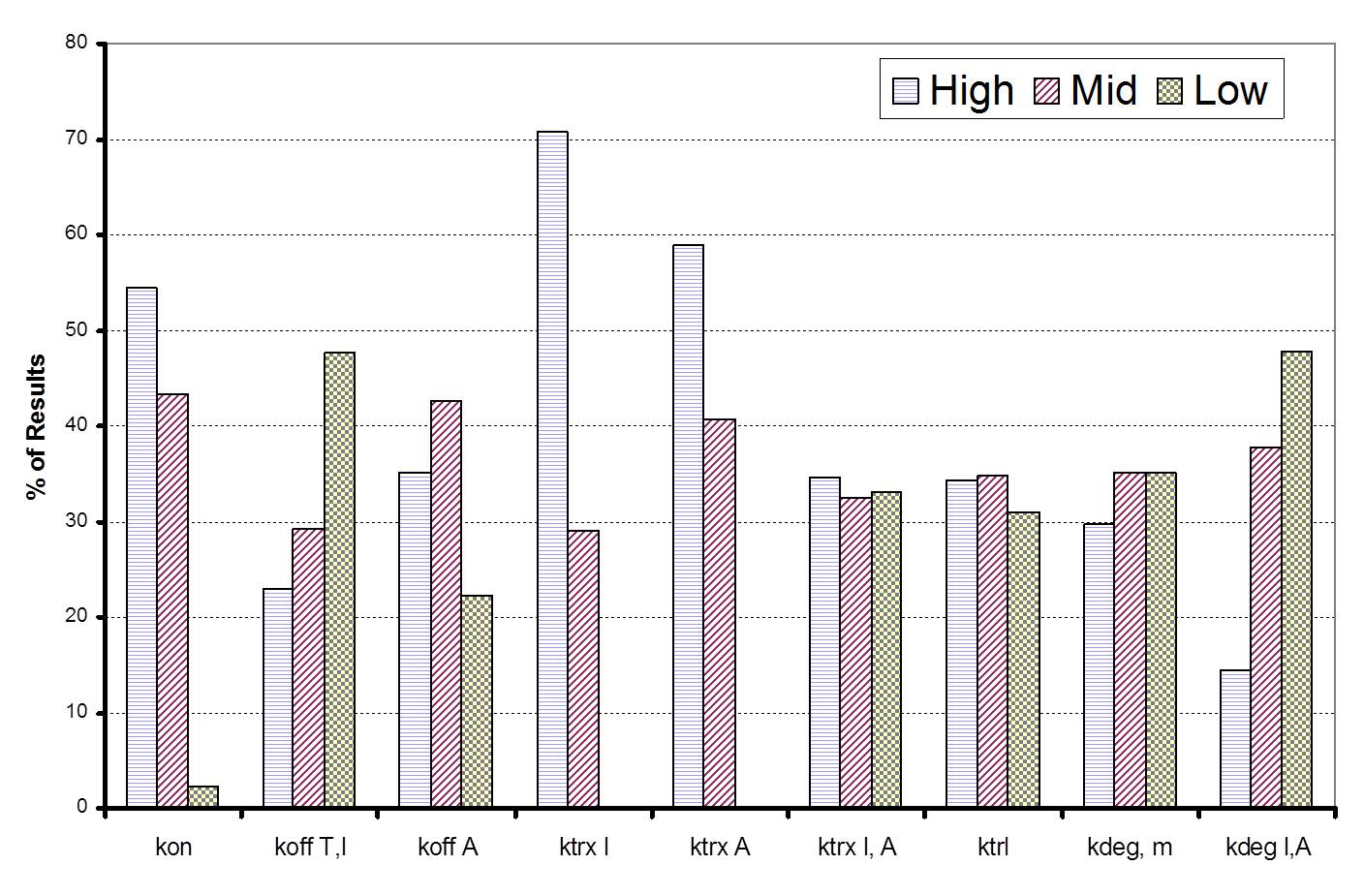

Supplement: Figure S1 — Depiction of sensitivity analysis results. Simulations were performed with all combinations of values (high, medium, low) for the nine different classes of parameters where the description of the classes and their respective high, medium, and low values are defined by Table S1. Each parameter set that led to a positive result (i.e. signal duration allows for a decision between I and A) was recorded. We computed for each of the 9 parameter classes, what percentage of these parameter sets had high values, medium values, or low values for the given parameter class. As an example, consider ktrx,I. Out of all the parameter sets that led to a positive result, 71% had the high value for ktrx,I, 29% had the medium value, and zero had the low value. (JPG) [file pone.0033018.s002.jpg]

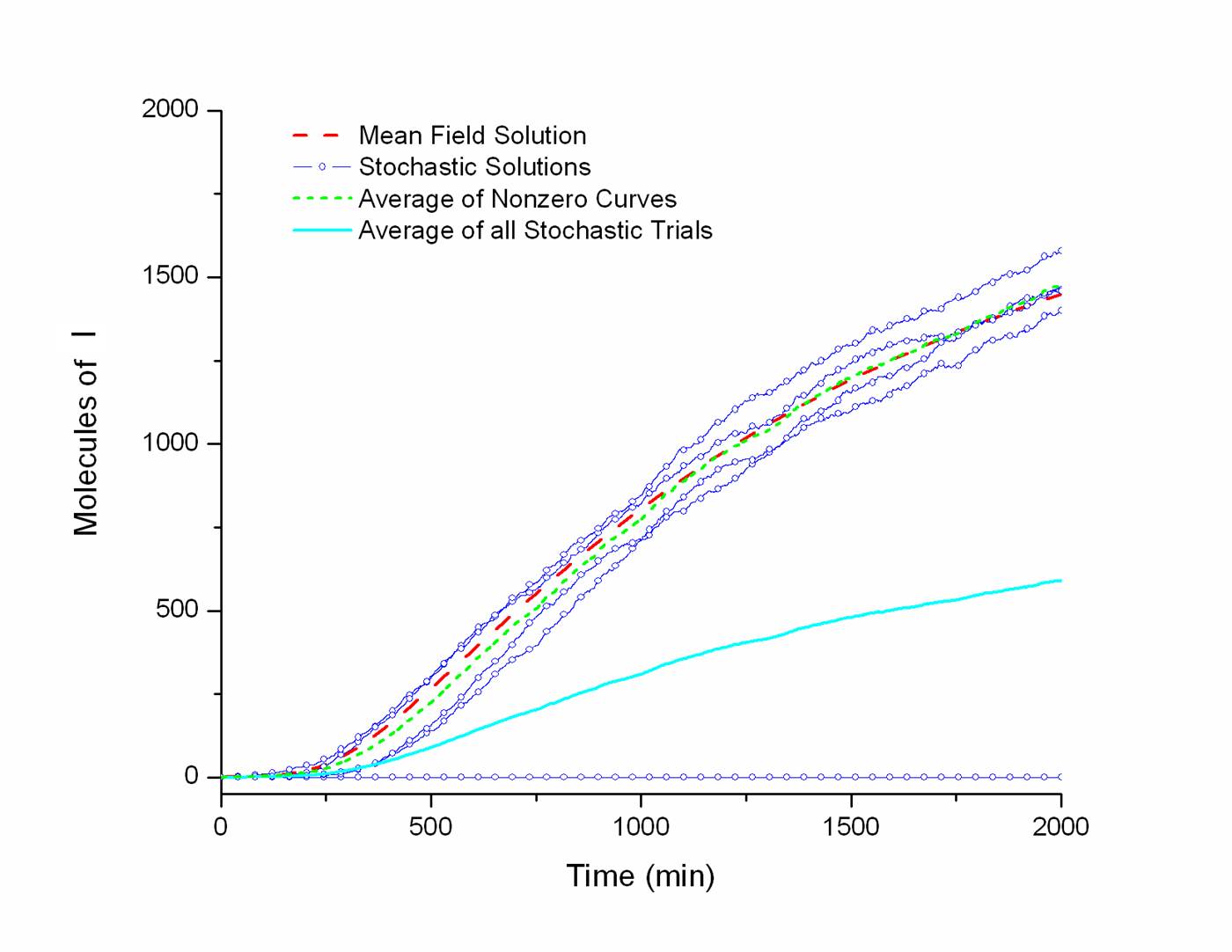

Supplement: Figure S2 — Only the nonzero stochastic solution trajectories follow the mean field solution to the model equations for molecule I at a signal degradation rate of 1.0 min−1. Time course trajectories obtained by solution of the Model 1 equations using a mean field ODE solver (red curve), ten times using the Gillespie Algorithm (10 blue curves), average at each time point of the nonzero stochastic trajectories (green curve), and average at each time point of all the stochastic trajectories (cyan curve). The results were generated using Model 1 and the parameters were obtained from Table 1. (JPG) [file pone.0033018.s003.jpg]

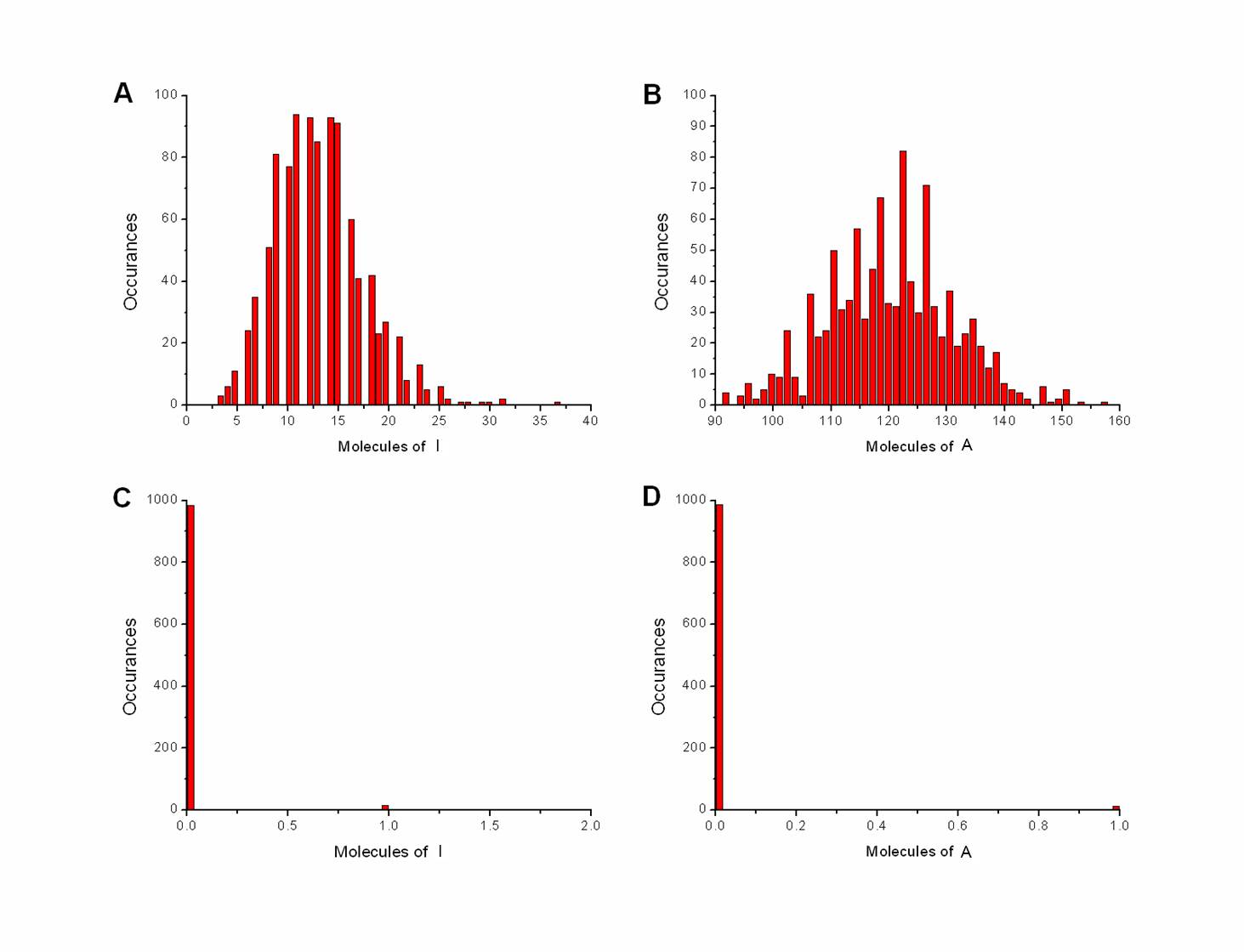

Supplement: Figure S3 — Upon deletion of the positive feedback loop, A is dominates transcription for sustained signals, while neither is significantly transcribed for transient signals. Histogram for (A) molecule I and (B) molecule A obtained by solving the Model 1 equations for a sustained signal of Kdeg,T equal to 0.0 min−1 using the Gillespie Algorithm 1000 times and recording the number of molecules at 2000 min. Histogram for (C) molecule I and (D) molecule A obtained by solving the Model 1 equations for a transient signal of Kdeg,T equal to 1.0 min−1 using the Gillespie Algorithm 1000 times and recording the number of molecules at 2000 min. Parameters for Model 1 were obtained from Table 1. (JPG) [file pone.0033018.s004.jpg]

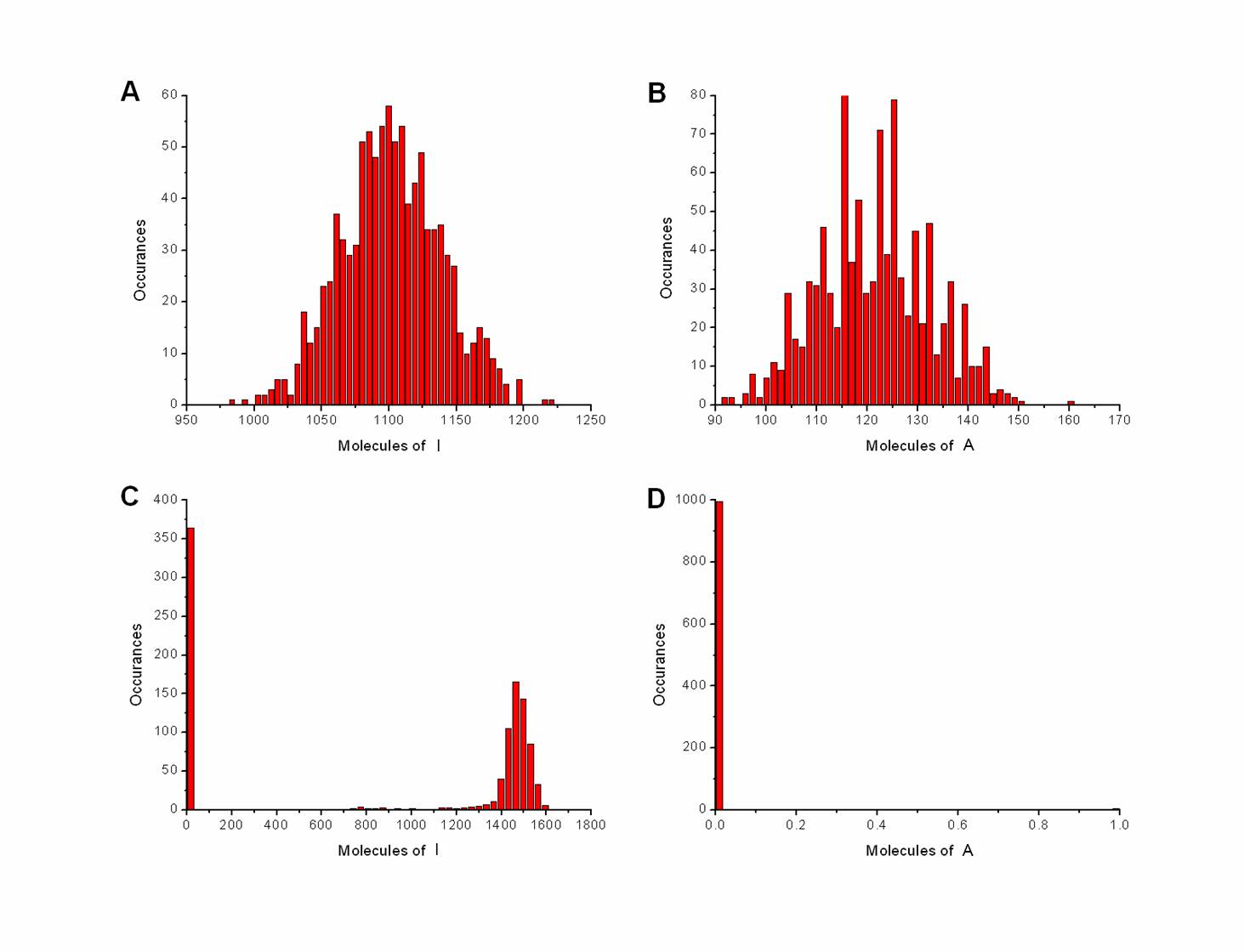

Supplement: Figure S4 — Upon deletion of the negative feedback loop, both I and A are strongly transcribed for sustained signals, while only I is significantly transcribed for transient signals. Histogram for (A) molecule I and (B) molecule A obtained by solving the model equations for a sustained signal of Kdeg,T equal to 0.0 min−1 using the Gillespie Algorithm 1000 times and recording the number of molecules at 2000 min. Histogram for (C) molecule I and (D) molecule A obtained by solving the Model 1 equations for a transient signal of Kdeg,T equal to 1.0 min−1 using the Gillespie Algorithm 1000 times and recording the number of molecules at 2000 min. Parameters for the model were obtained from Table 1. (JPG) [file pone.0033018.s005.jpg]

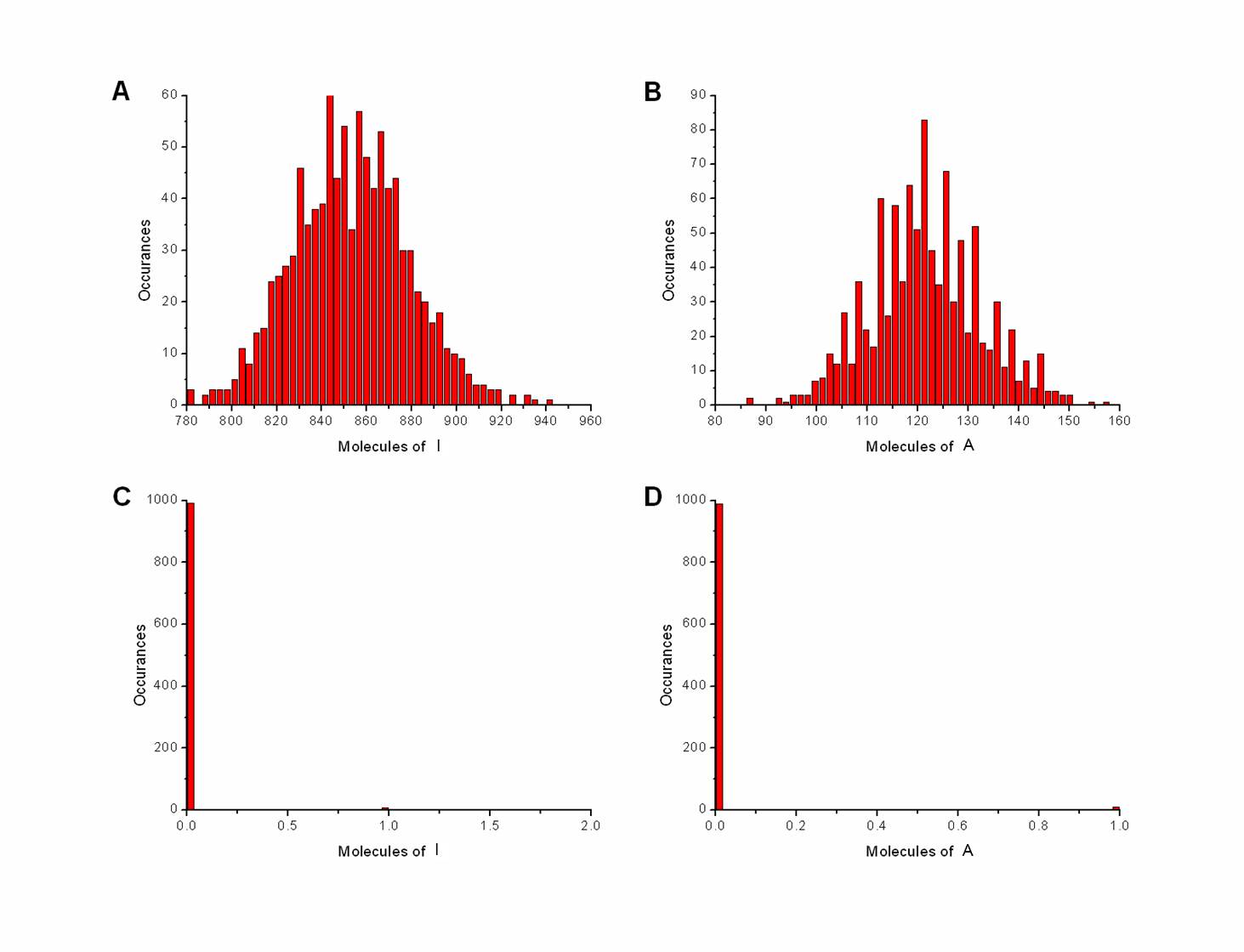

Supplement: Figure S5 — Upon deletion of the positive and negative feedback loops, both I and A are produced in significant quantities after 2000 min for sustained signals, while neither is produced in significant quantities for transient signals. Histogram for (A) molecule I and (B) molecule A obtained by solving the Model 1 equations for a sustained signal of Kdeg,T equal to 0.0 min−1 using the Gillespie Algorithm 1000 times and recording the number of molecules at 2000 min. Histogram for (C) molecule I and (D) molecule A obtained by solving the Model 1 equations for a transient signal of Kdeg,T equal to 1.0 min−1 using the Gillespie Algorithm 1000 times and recording the number of molecules at 2000 min. Parameters for the model were obtained from Table 1. (JPG) [file pone.0033018.s006.jpg]

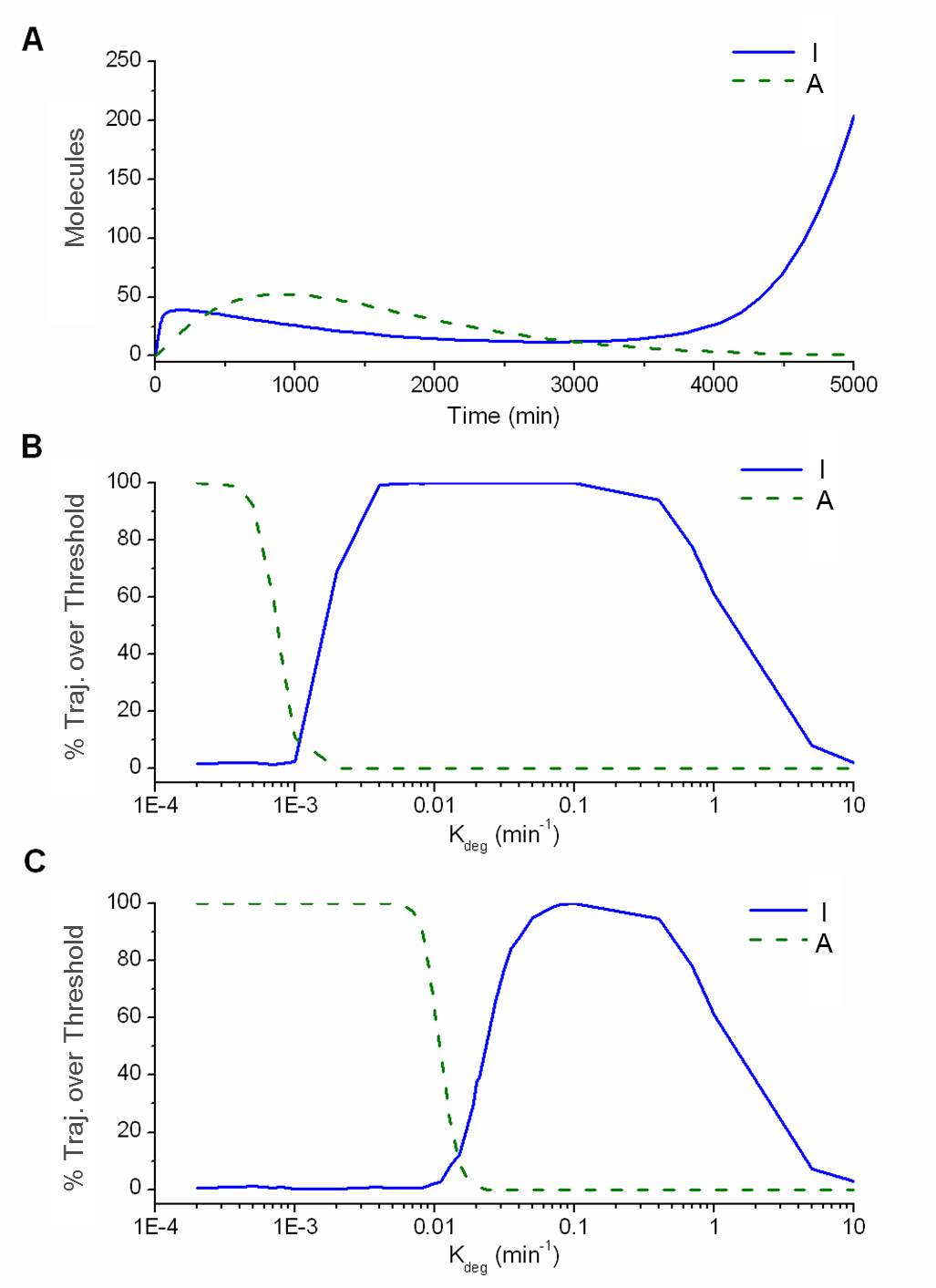

Supplement: Figure S6 — At very long times the model breaks down and I dominates transcription for all nonzero signals. This is due to the fact that A is entirely signal dependent, so once the signal is gone it also decays to zero rendering it incapable of inhibiting the production of I. Since I has not decayed to zero, its positive feedback loop allows it to ramp up production when A is incapable of inhibition. (A) Mean field solution for molecule I at a signal degradation rate of 0.002 min−1 plotted out to 5000 min. (B) The maximum amount of I and A observed in 1000 time course trajectories measured out to 5000 min was compared to an arbitrary threshold and the percentage of trajectories crossing the threshold was computed for a wide range of signal degradation rates. The I and A thresholds were set to 100 molecules. The results were generated using Model 1 and the parameters were obtained from Table 1, thus Kdeg,A is equal to 0.001 min−1. (C) Reducing the degradation rate of A (Kdeg,A) to a value of 0.0001 min−1 counteracts the effect of measuring out to 5000 minutes. The I threshold was set to 100 molecules, while the A threshold was set to 20 molecules. Sampled trajectories were 5000 minutes long. (JPG) [file pone.0033018.s007.jpg]
