# Supplementary material for: A Model for How Signal Duration Can Determine Distinct Outcomes of Gene Transcription Programs
Source: PLoS One. 2012 Mar 13;7(3):e33018. doi: 10.1371/journal.pone.0033018 (PMC3302786; doi:10.1371/journal.pone.0033018)
Supplement: Table S1 — Listing of the parameter classes and their corresponding low, mid, and high values used in the sensitivity analysis. Low, mid, and high values for each parameter were selected arbitrarily to either test over a range of orders of magnitude or to test at values around those used to obtain all the results in the main text (Table 1 parameter values). (DOC) [file pone.0033018.s008.doc]

| Parameter Class | Description | Low | Mid | High |
| --- | --- | --- | --- | --- |
| k_on_ (molec^-1^ min^-1^) | On-rate for all molecules on all genes | 0.0015 | 0.015 | 0.15 |
| k_off A,B_ (min^-1^) | Off-rate of A and B | 0.01 | 0.5 | 1.0 |
| k_off C_ (min^-1^) | Off-rate of C from gene B | 0.01 | 0.5 | 1.0 |
| k_trx B_ (min^-1^) | Transcription rate of B in the absence of C | 0.01 | 0.1 | 1.0 |
| k_trx C_ (min^-1^) | Transcription rate of C | 0.01 | 0.1 | 1.0 |
| k_trx B,C_ (min^-1^) | Transcription rate of B in the presence of C | 0 | 0.001 | 0.009 |
| k_trl_ (min^-1^) | Translation rate of B and C | 0.01 | 0.5 | 1.0 |
| k_deg,m_ (min^-1^) | mRNA degradation rate | 0 | 0.001 | 0.01 |
| k_deg B,C_ (min^-1^) | Degradation rate of B and C | 0 | 0.001 | 0.01 |

Table S1. Listing of the parameter classes and their corresponding low, mid, and high values used in the sensitivity analysis. Low, mid, and high values for each parameter were selected arbitrarily to either test over a range of orders of magnitude or to test at values around those used to obtain all the results in the main text (Table 1 parameter values).
