# Supplementary material for: A Model for How Signal Duration Can Determine Distinct Outcomes of Gene Transcription Programs
Source: PLoS One. 2012 Mar 13;7(3):e33018. doi: 10.1371/journal.pone.0033018 (PMC3302786; doi:10.1371/journal.pone.0033018)
Supplement: Table S2 — Table of parameters used in Model 2. This is the model where transcription factors do not dissociate after a transcriptional event. The parameters were selected in order to clearly demonstrate the phenomena of interest (i.e. I dominates transcription for transient signals while A dominates for sustained signals). (DOC) [file pone.0033018.s009.doc]

| k_on,T,A_ | 0.015 molec^-1^ min^-1^ | k_trx,I,A_ | 0 min^-1^ |
| --- | --- | --- | --- |
| k_on,I,I_ | 0.015 molec^-1^ min^-1^ | k_trl,I_ | 1.0 min^-1^ |
| k_on,A,I_ | 0.015 molec^-1^ min^-1^ | k_trl,A_ | 1.0 min^-1^ |
| k_off,T,I_ | 0.5 min^-1^ | k_deg,T_ | 0-10 min^-1^ |
| k_off,T,A_ | 0.5 min^-1^ | k_deg_ | 0.01 min^-1^ |
| k_off,I,I_ | 0.5 min^-1^ | k_deg,m_ | 0.01 min^-1^ |
| bk_off,A,I_ | 0.5 min^-1^ | pSTAT3_o_ | 100 molec. |
| k_trx,I,T_ | 1.0 min^-1^ | I_g_ | 2 molec. |
| k_trx,A,T_ | 1.0 min^-1^ | A_g_ | 2 molec. |

**Table S2. Table of parameters used in Model 2.** This is the model where transcription factors do not dissociate after a transcriptional event. The parameters were selected in order to clearly demonstrate the phenomena of interest (i.e. I dominates transcription for transient signals while A dominates for sustained signals).
